# A systematic screen for morphological abnormalities during fission yeast sexual reproduction identifies a mechanism of actin aster formation for cell fusion

**DOI:** 10.1371/journal.pgen.1006721

**Published:** 2017-04-14

**Authors:** Omaya Dudin, Laura Merlini, Felipe O. Bendezú, Raphaël Groux, Vincent Vincenzetti, Sophie G. Martin

**Affiliations:** Department of Fundamental Microbiology, University of Lausanne, Lausanne, Switzerland; University of California San Francisco, UNITED STATES

## Abstract

In non-motile fungi, sexual reproduction relies on strong morphogenetic changes in response to pheromone signaling. We report here on a systematic screen for morphological abnormalities of the mating process in fission yeast *Schizosaccharomyces pombe*. We derived a homothallic (self-fertile) collection of viable deletions, which, upon visual screening, revealed a plethora of phenotypes affecting all stages of the mating process, including cell polarization, cell fusion and sporulation. Cell fusion relies on the formation of the fusion focus, an aster-like F-actin structure that is marked by strong local accumulation of the myosin V Myo52, which concentrates secretion at the fusion site. A secondary screen for fusion-defective mutants identified the myosin V Myo51-associated coiled-coil proteins Rng8 and Rng9 as critical for the coalescence of the fusion focus. Indeed, *rng8Δ* and *rng9Δ* mutant cells exhibit multiple stable dots at the cell-cell contact site, instead of the single focus observed in wildtype. Rng8 and Rng9 accumulate on the fusion focus, dependent on Myo51 and tropomyosin Cdc8. A tropomyosin mutant allele, which compromises Rng8/9 localization but not actin binding, similarly leads to multiple stable dots instead of a single focus. By contrast, *myo51* deletion does not strongly affect fusion focus coalescence. We propose that focusing of the actin filaments in the fusion aster primarily relies on Rng8/9-dependent cross-linking of tropomyosin-actin filaments.

## Author summary

Sexual reproduction is a common process in most eukaryotic species. In those with non-motile gametes, such as most fungi, important morphological changes underlie this process. We report on a systematic screen for mutants with morphological abnormalities during sexual reproduction in the fission yeast, *Schizosaccharomyces pombe*, a widely used model for the study of fundamental cellular processes. The results of this screen expose a host of novel gene functions required for cell polarization, cell-cell fusion and production of progeny through sporulation. We then focused on the large class of genes required for the fusion of the two gametes into a single zygote. Cell fusion requires the assembly of an intracellular actin cytoskeleton-based structure, named the fusion focus, which concentrates the delivery of enzymes for cell wall digestion to a precise site. We show that fusion focus coalescence into a single aster requires the action of the Rng8/9 dimer. Our work indicates that cross-linking of tropomyosin-decorated actin filaments by the Rng8/9 dimer is critical to actin filament focusing.

## Introduction

Sexual reproduction is carried out by most eukaryotes and permits the alternation of haploid and diploid life stages. It relies on the formation of differentiated haploid cell types that are able to meet and fuse to form a zygote, which eventually returns to the haploid state through meiosis. Many of these events rely on morphological changes, especially in organisms without cell motility. Yeast model systems have been used over decades to uncover basic principles of cell organization, yet no systematic screening of their sexual reproduction process has been performed. Here, we have used the fission yeast *Schizosaccharomyces pombe* to systematically screen for viable gene deletions causing a morphological abnormality in the sexual reproduction process. We anticipated this screen would shed light on the processes of cell polarization, cell-cell fusion and sporulation.

All natural *S*. *pombe* isolates live as haploid cells, and many, such as the *h90* lab strain, are self-fertile (homothallic) [1,2]. These cells, which can be of two distinct mating types, P and M, regularly switch mating type by recombination of the silent mating cassette into the active site after cell division, thus resulting in a near genetically identical population that can reproduce sexually [3]. Sexual differentiation is initiated by nitrogen starvation, which leads to the expression of pheromones and cognate receptor on the two cell types. Pheromone signaling involves a GPCR-MAPK signal transduction cascade, which in turn reinforces sexual differentiation and initiates the morphological program of mating [4]. Upon sensing low pheromone levels, cells initially polarize secretion towards a cortical patch assembled around the active form of the small GTPase Cdc42 [5]. This patch dynamically forms at various cortical locations and disassembles over time, but cells do not grow. Pheromone secretion and sensing are thought to occur at the patch, which is stabilized through unknown molecular mechanisms upon higher local pheromone perception, such that two neighboring cells become locked together when their patches meet [6]. Paired cells then grow towards each other to form a pre-zygote, with cell wall still separating the two partner cells.

To achieve cell fusion, the cell wall needs to be digested at the zone of cell-cell contact to allow plasma membrane fusion. This relies on the fusion focus, a dedicated actin aster nucleated by the formin-family protein Fus1, which promotes the convergence on a small cortical zone of secretory vesicles transported by type V myosin motors [7–9]. In particular, these motors transport glucanases, enzymes that hydrolyze the bonds linking the cell wall glucan polymer [7]. Over the course of the fusion process, the fusion focus forms from an initially broad distribution at the cell projection tip, and stabilizes into a single focus in opposing locations in the two partner cells. This stabilization stems from a positive feedback between concentration of pheromone signal at the secretion zone and local enrichment of the pheromone signal transduction machinery, which immobilizes the fusion focus through unknown mechanism [10]. In turn, spatial stabilization permits the focused delivery of glucanases for local cell wall digestion.

Formation of the fusion focus is likely to require several actin-binding proteins, in addition to Fus1. In particular, profilin Cdc3 and tropomyosin Cdc8 are enriched on the structure and necessary for cell-cell fusion [11,12]. Type V myosins also localize on the fusion focus and contribute to its focalization [7,13]. There are two such myosins in fission yeast [14,15]: Myo52 is the main cargo transporter for both cell polarization and cell fusion [7,16–18], and moves processively on tropomyosin-decorated actin filaments [19]; Myo51 is more unusual, as many of its functions are independent of its cargo-binding tail [7,20,21]. In addition, Myo51 is a single-headed motor protein, and both in vivo and in vitro experiments have shown that only motor ensembles were capable of processive movement [21,22]. In vivo, a dimer of two coiled-coil proteins, Rng8 and Rng9, associates with Myo51, regulates its localization during mitotic growth, and was proposed to contribute to Myo51 processivity by forming higher-order oligomers in vivo [21]. In vitro, the Rng8/9-Myo51 complex was also shown to bind tropomyosin-decorated F-actin independently of the motor domain, thus forming a bivalent F-actin-binding complex cross-linking and sliding actin-tropomyosin filaments relative to one another [22]. Despite these recent advances, how these motors or other actin-binding proteins function to focus an actin aster is not established.

Upon local cell wall digestion, plasma membranes fuse. Though multi-pass transmembrane proteins such as Prm1 have been suggested to participate in this process in several fungal species, the mechanism remains completely elusive [23–25]. As the fusion pore then expands, the neck connecting the now fused cells is remodeled to create an elongated zygote in which the two parental haploid nuclei fuse. The diploid nucleus then enters meiosis to return the genome to its haploid state, forming four meiotic products, each of which is packaged in a stress-resistant spore. Sporulation is a very morphologically demanding process in which new plasma membrane and new wall is laid down, initiated from the spindle pole associated with each of the four genomic meiotic products [26].

Previous forward-genetic screens have identified a number of sterile, fusion-defective and sporulation-deficient mutants, and a targeted genome-wide screen for sporulation-defective deletion strains was published in the course of this work [27]. However, there has not been any systematic reverse-genetic screen of the mating process. Here, we present the results of a visual screen for morphological abnormalities during the mating process in fission yeast. Our screen led us to identify the Rng8/9 dimer and its interaction with tropomyosin as critical for the formation of the actin fusion focus. We propose that cross-linking of tropomyosin-actin filaments serves to focalize filaments in the fusion focus.

## Results

### Creation and visual screening of a homothallic deletion collection

**Fig 1:**
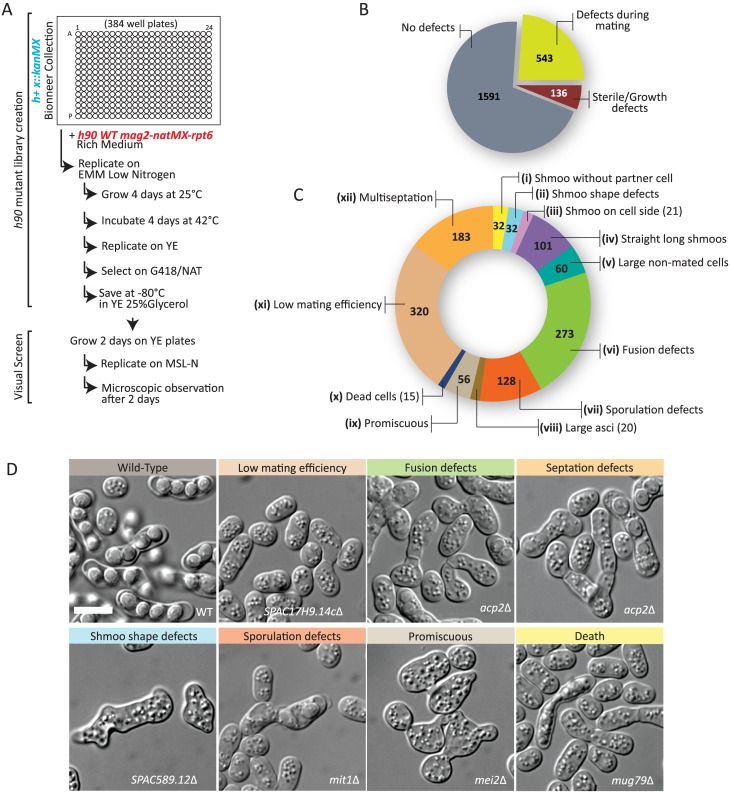
Creation of a homothallic deletion library and visual screening during mating. (A) Workflow used to create the homothallic *h90* deletion library and to visually screen it for morphological abnormalities during mating. (B) Pie chart representing the total number of deletion strains identified to display a visible phenotype during mating. Note that strains noted as “no defect” contain those assigned to the *multiseptation* and *low mating efficiency* classes, which may not be mating-specific. (C) Distribution of all 543 deletion strains with a phenotype in the described phenotypic classes. Note that the total number is >543 because 1) the mutants displaying low mating efficiency and multiseptation phenotypes are also included, and 2) some deletion strains display several phenotypes. (D) Representative images of phenotypic classes. Bars, 5 μm.

To systematically screen the collection of viable deletions for mating defects, we converted the available heterothallic *h+* deletion library [28] to a homothallic *h90* collection by applying a modified version of the SpSGA method [29]. We first integrated a nourseothricin resistance cassette (*natMX*) 6kb away from the expressed *mat1* mating-type cassette, between the genes *mag2* and *rpt6*, in an otherwise wildtype homothallic *h90* strain. Because the genomic region located between the expressed *mat1* locus and silent *mat* loci represents a genetic distance of only 1cM [30], the *h90* trait and *natMX* largely co-segregate, allowing for selection for the *h90* trait at the population level. We then robotically crossed this *h90-natMX* strain to all *kanMX*-marked deletion strains of the *h+* collection in 384-well plate format. Mating was induced on solid medium with low nitrogen for 4 days at 25°C. Vegetative haploid cells that had not mated and diploid cells that had not sporulated were killed by incubation at 42°C for 4 days [29]. We note that diploid killing was efficient, as azygotic tetrads, which stem from the sporulation of diploid cells rather than zygotes formed by cell-cell fusion, were observed in only 76/2270 strains upon the visual screening described below. Spore germination was triggered by replica plating on solid rich medium (YE). A second replication step to solid medium containing both G418 and nourseothricin selected for homothallic *h90* deletion-carrying strains. Finally, strains were saved at -80°C in YE 25% glycerol (Fig 1A).

From 2270 deletion strains of the *h+* deletion collection, we recovered 2134 *h90* derivatives. The 136 that we could not recover are likely to be either sterile (for example *ste4*Δ, *ste6Δ*, *ste7Δ*, *ste20Δ*, *ras1Δ*, *wee1Δ*) or too sick to have efficiently crossed, and thus did not give spore progeny in the scheme above. We did not investigate those further at present. To find mutants affecting the mating process, we visually screened the *h90* mutants after a 2-day incubation on solid medium lacking nitrogen (MSL-N). The visual screen was performed in replica by two independent investigators with deletion names undisclosed, in order to eliminate any bias.

Remarkably, out of the 2134 screened mutants, 543 mutant showed a visible phenotype during the mating process (Fig 1B). Ten distinct phenotypes were recorded: these included early mating polarization defects, such as (i) the presence of cells extending growth projections not meeting a partner cell, (ii) aberrant shmoo shapes, (iii) placement, or (iv) length, or (v) the presence of abnormally large unmated cells; (vi) fusion defects, in which paired cells were observed with cell wall at the contact site; and post-fusion phenotypes, such as (vii) sporulation defects, in which asci had abnormal spore numbers or shapes, (viii) abnormally large asci, or (ix) promiscuous cells, in which mutants appeared to mate (or attempt to mate) with multiple partners. Finally, we recorded (x) the presence of dead cells in the mating assay, which may be caused by cell lysis upon deregulated fusion attempts [10]. In addition to these ten classes, we assigned mutants in which cell pairs were rare and/or individual cells did not appear to be arrested as small cells to a (xi) *low mating efficiency* class. We also scored for (xii) multiseptation, in which cells showed multiple septa, though this phenotype may not be starvation-specific. For each of these categories, the severity of the phenotype was gauged on a scale from 1 to 10. We note that some deletions were labeled with several distinct phenotypes. A summary of these categories, with the number of identified mutants, is represented in Fig 1C. Representative images for some phenotypic classes are shown in Fig 1D. The full description of each phenotypic class, as well as the complete table of mutants with their recorded phenotypes, is available as supplementary material (S1 and S2 Tables).

### Fusion-deficient mutants

**Fig 2:**
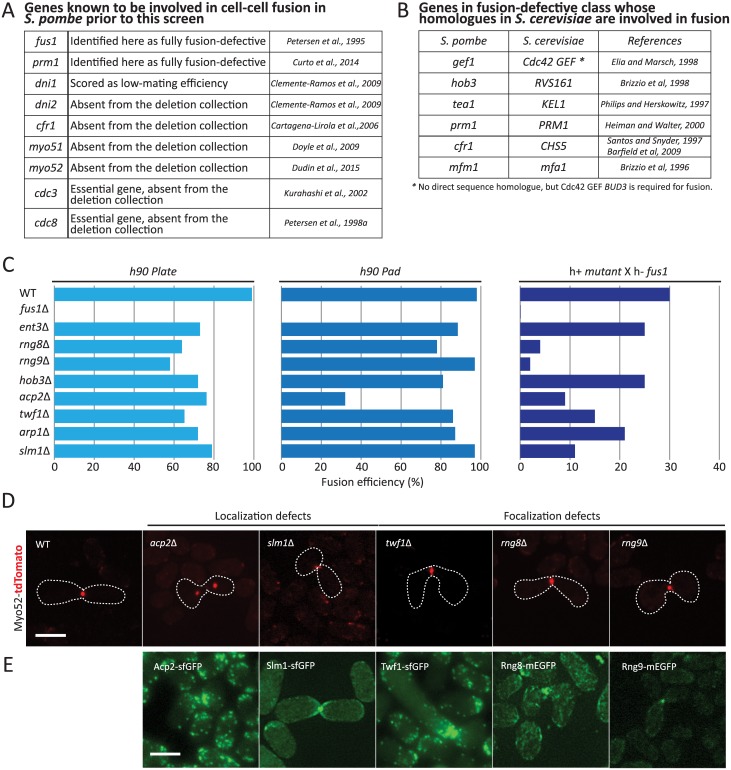
Fusion deficient mutants. (A) List of all *S*. *pombe* genes known to be involved in cell-cell fusion prior to this screen. (B) List of genes identified to be required for cell fusion with homology to *S*. *cerevisiae* genes involved in fusion. (C) Fusion efficiency of selected homothallic mutants on plate (left), on a pad between slide and cover-slip (middle) and of heterothallic mutants crossed to *fus1Δ* on a pad. (D) Homothallic mutants expressing Myo52-tdTomato. Images shown are maximum intensity projections of a time-series of 7 z-stacks over 15 seconds, except for *slm1Δ*, where a maximum intensity projection of one single time point is shown, which illustrates better the imprecise position of the fusion focus. Compare to wildtype in Fig 3A and 3C. (E) Maximum intensity projection images of *h90* wild-type strains expressing Acp2-GFP, Slm1-mEGF, Twf1-GFP, Rng8-mEGFP and Rng9-mEGFP respectively, during fusion. Bars, 5 μm.

We focused our analysis on the *fusion defects* class of 273 mutants affecting the cell-cell fusion process (S3 Table). We compared these mutants with a list of genes involved in cell-cell fusion compiled from the literature (Fig 2A). As expected, we identified *fus1Δ* and *prm1Δ* as fusion defective [9,23]. Deletions of *myo51*, *myo52* and *cfr1* have also been shown to lead to fusion defects [7,13,31], but these strains were absent from the screened library, as were of course deletion of the essential tropomyosin Cdc8 and profilin Cdc3, also required for fusion [11,12]. We also did not identify *dni1Δ* and *dni2Δ*, likely because these genes are required for fusion only at elevated temperatures [32]. This suggests our screen identified all of the identifiable, previously known genes involved in cell fusion. Comparison of our list of fusion-defective mutants also identified several homologues to *S*. *cerevisiae* cell fusion factors (Fig 2B; see Discussion). Amongst all deletions with an arbitrary score of 3 or above, we performed a GO Slim analysis of the gene products, which revealed that 12.5% are components of the cytoskeleton, an enrichment relative to the 7.4% within the whole genome.

The enrichment of cytoskeletal components in fusion-defective mutants is interesting because fusion relies on a dedicated aster-like actin structure, the fusion focus [7,10]. To further explore novel fusion-defective mutants affecting the cytoskeleton, we first used publically available data (pombase.org) to discard from the list of cytoskeleton components mutants implicated in chromatin remodeling, spore formation, or with a known localization in the nucleus. This left us with 8 fusion-defective, cytoskeleton-related mutants (Fig 2C), amongst which was the pheromone-dependent formin Fus1 [9]. All 8 strains were verified by PCR for correct deletion of the corresponding gene. The others include the actin capping protein Acp2, previously involved in actin cytoskeleton organization during mitotic growth [33,34], the centractin family actin like protein Arp1, part of the dynactin complex previously implicated in dynein-dependent nuclear movement during meiotic prophase (horsetail movement; [35]), the actin monomer-binding protein twinfilin *twf1* involved in regulation of polarized growth [36], and the BAR-domain protein Hob3, which was previously known to regulate cytokinesis in part through regulation of Cdc42 GTPase [37]. A recently described regulator of the type V myosin Myo51, Rng8, was also selected [21]. We also included Rng9, the Rng8 binding partner [21], in the subsequent analysis although it was not identified in the screen.

To monitor the fusion deficiency of the selected mutants, we used two distinct assays. First, we reproduced the screen conditions by placing cells on MSL-N plates for 24h and counting the percentage of non-fused pairs after transfer to a microscope slide (Fig 2C, left). In this three-dimensional assay, multiple layers of cells are able to mate with each other. We also used our previously established protocol to quantify fusion efficiency after 24 hours on MSL-N agarose pads, where cells are trapped in a two-dimensional monolayer for the duration of the sexual reproduction (Fig 2C, middle) [38]. While only *fus1Δ* was fully fusion-defective, all mutants showed some fusion defect in at least one of the two assays. We note that, with one exception, the fusion defect was more severe in the three-dimensional assay. This may be due to differences in pheromone distribution or oxygen availability between the two conditions. We also investigated the fusion efficiency on pads of the selected mutants in a heterothallic background with a *fus1Δ* partner, which is fully fusion-deficient. This more stringent test assesses the capacity of the mutant to overcome the fusion deficiency of its partner cell. In this set-up, again all mutants were more fusion-defective than wildtype cells, with 4 mutants highly fusion defective (fusion efficiency < 20%): *rng8*Δ, *acp2*Δ, *twf1*Δ and *slm1*Δ (Fig 2C, right). Deletion of *rng9* yielded a similar phenotype as *rng8Δ*.

**Fig 3:**
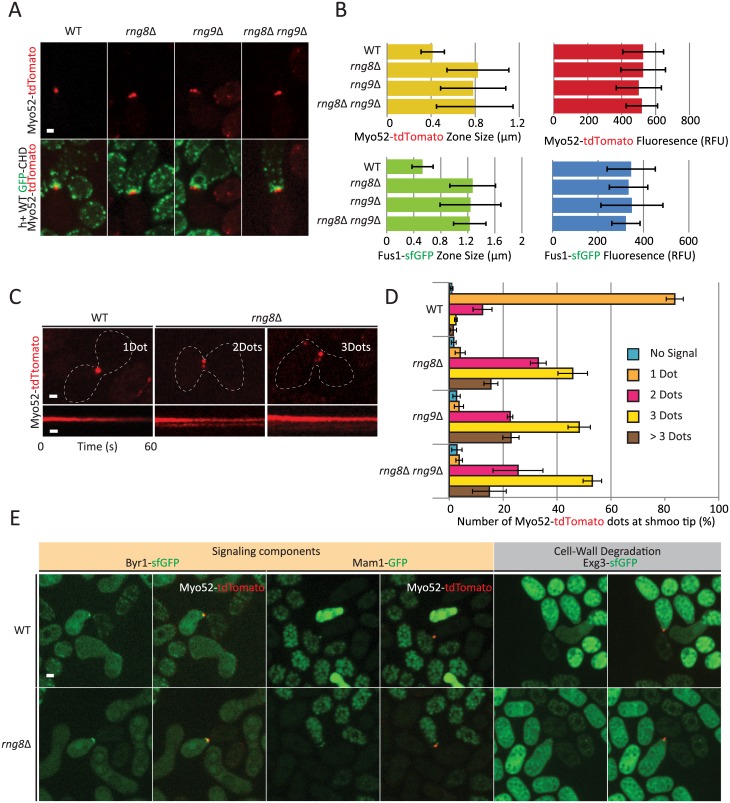
Rng8 and Rng9 are required for the formation of a single fusion actin focus. (A) Cross of wildtype *h+* strain expressing the F-actin marker GFP-CHD with *h-* wildtype, *rng8*Δ, *rng9*Δ or double *rng8*Δ *rng9*Δ mutant strains expressing Myo52-tdTomato, showing a more dispersed Myo52-tdTomato signal in the mutant strains. Images are maximum intensity projection of a time-series of 7 z-stacks over 15 seconds. (B) Measurements of Myo52-tdTomato and Fus1-sfGFP zone width and fluorescence intensity at the cell-cell contact site. Measurements were done on sum projections of 7 z-stacks. (N = 15). (C) Typical kymographs of Myo52-tdTomato showing multiple stable dots over >60s in *rng8*Δ in comparison to the unique fusion focus in wildtype. (D) Quantifications of the number of Myo52-tdTomato dots observed in wildtype, *rng8*Δ, *rng9*Δ and *rng8*Δ *rng9*Δ mutants. The small percentage of cells showing two or more dots in wildtype likely represents cells captured in the process of forming the fusion focus (see also S2 Fig) (N>100 cells). (E) Cross of wildtype *h+* cells expressing Myo52-tdTomato with *h-* wildtype or *rng8*Δ expressing Byr1-GFP, Mam1-GFP or Exg3-GFP and Myo52-tdTomato. Images shown are maximum intensity projection of a time-series of 7 z-stacks over 15 seconds. Arrowheads point at cells in fusion. Bars, 2 μm.

All five deletion strains displayed defects in fusion focus organization, as labeled with Myo52-tdTomato (Fig 2D): *acp2Δ* and *slm1Δ* displayed aberrant localization of the fusion focus, with the focus often detached from the cell projection tip in *acp2Δ* and out of alignment in *slm1Δ*. *rng8Δ*, *rng9Δ and twf1Δ* showed wider Myo52-tdTomato signals (see Fig 3A and 3B for quantifications), suggestive of a defect in fusion focus focalization. Fluorescence tagging of each of the five genes at endogenous locus revealed that all accumulated at the fusion site, albeit with different localization patterns. Acp2 and Twf1 appeared to primarily decorate actin patches as previously shown for Acp2 during vegetative growth [34]. Slm1 was highly concentrated at the fusion focus, but was also decorating the cortex of the entire projection tip. By contrast, Rng8 and Rng9 accumulated in a concentrated location at the fusion site (Fig 2D), which coincided with the Myo52-labelled fusion focus (S1 Fig).

In summary, we successfully identified several new genes affecting the fusion process in fission yeast. As all five deletion strains above readily reveal a defect in fusion focus organization, and all encoded proteins localize at the fusion site, we conclude that the screen was highly successful in detecting genes directly involved in the regulation of cell fusion. By extension, this also suggests that many other fusion-defective deletion strains will also reveal interesting new cell fusion phenotypes.

### Rng8 and Rng9 are crucial for focalization of the fusion focus

Because Rng8 and Rng9 localize to the fusion focus and appear to be required for its focalization, we extended our analysis of their function for the dynamics of the fusion focus during the fusion process, using high-temporal resolution time-lapse microscopy. In *rng8Δ*, *rng9Δ* and double *rng8Δ rng9Δ* mutants mated with wildtype cells, the major fusion focus components Myo52-tdTomato and formin Fus1-sfGFP occupied a zone about twice as wide as in wildtype cells, when measured on sum Z-projections, though the total signal detected at the cell-cell contact site was unchanged (Fig 3A and 3B). Time-lapse imaging of Myo52-tdTomato in single focal planes further showed that *rng8Δ*, *rng9Δ* and double *rng8Δ rng9Δ* mutants display multiple stable Myo52 dots at the shmoo tip (Fig 3C and 3D). Whereas two dots are occasionally observed in wildtype cells as the fusion focus forms when the signal matures from a broad crescent-like localization to a single dot (S2 Fig), we never observed several stable dots in wildtype cells. By contrast, in *rng8/9* mutants, most cells exhibited 2, 3 or more dots that were spatially stable over > 1 minute at the cell cortex (Fig 3C and 3D). This phenotype, as well as fusion efficiency (S3 Fig), were indistinguishable in single and double mutants, consistent with Rng8 and Rng9 forming an obligate dimer [21,22]. Our further analysis was thus conducted only on the *rgn8Δ* single mutant. We conclude that the Rng8/9 dimer is required for the formation of a single fusion focus structure.

Stabilization of the fusion focus relies on accumulation of the pheromone signaling machinery on the structure [10]. In wildtype cells, both M-factor transporter Mam1 and components of the pheromone transduction pathway, including the MAP2K Byr1, strongly accumulate on the fusion focus. In *rng8Δ* cells, these components were present at the fusion site, though over a wider region, similar to our description of Fus1 and Myo52 above (Fig 3E). Because of weaker signal intensity, we were unable to confidently determine whether Byr1 and Mam1 also systematically form several distinct stable dots, or have a more continuous, broad localization, though in some instances, several dots of Mam1 could be clearly distinguished (Fig 3E). This suggests each dot becomes stabilized through the normal pheromone signaling-dependent pathway [10]. This is consistent with the idea that Rng8 is required not for the immobilization of the fusion focus, but for the coalescence of the actin aster to a single structure prior to stabilization.

The fusion focus serves for the local release of cell wall digestive enzymes [7]. In wildtype cells, the glucanase Exg3-sfGFP can be clearly observed at the fusion focus. In *rng8Δ* cells, Exg3 could also be detected at the fusion site (Fig 3E), but only in about half of the cells and often over a wider zone, consistent with the idea that this glucanase is secreted over a broader region upon fusion focus coalescence defects. This defect is consistent with the lower efficiency of *rng8Δ* cells in digesting their cell wall, especially when mated with *fus1Δ* partners (Fig 2D). We conclude that the Rng8/9 dimer is critical for the coalescence of the acto-myosin fusion focus into a single aster-like structure, required for local release of cell wall digestive enzymes.

### Rng8 and Rng9 have roles beyond that of regulating Myo51 motor

Previous work has implicated the Rng8/9 dimer in the regulation of the single-headed myosin Myo51. Indeed, Rng8/9 associates with Myo51 in vivo and in vitro and promotes Myo51 cluster formation, Myo51 is not detected on actin cables and only very weakly at the cytokinetic ring in *rng8Δ* and *rng9Δ* cells, and these and *myo51Δ* mutants have similar defects in contractile ring assembly [21,22]. One proposed model is that Rng8/9 forms an integral part of the Myo51 motor for most or all of its cellular functions and is strictly required for its processivity [21]. We thus examined in detail the phenotype and localization of Myo51 during cell fusion.

**Fig 4:**
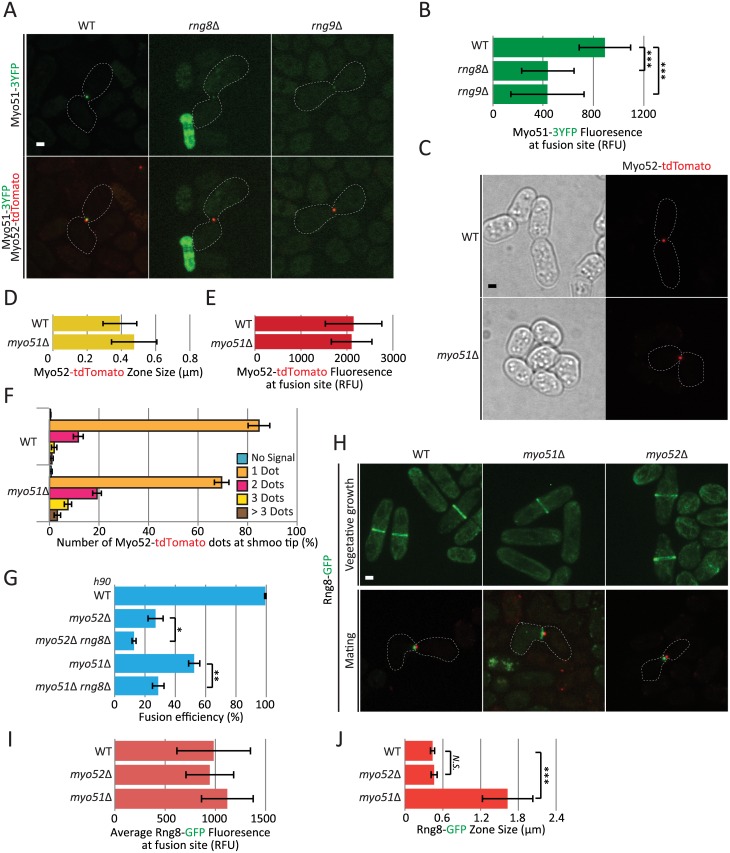
Rng8 and Rng9 have roles beyond that of regulating Myo51 motor. (A) Cross of wildtype *h-* expressing Myo52-tdTomato with *h+* wildtype, *rng8*Δ or *rng9*Δ strains expressing Myo51-3YFP. (B) Quantification of Myo51-3YFP intensity at the fusion site in wildtype, *rng8*Δ and *rng9*Δ strains as in (A), showing reduction of Myo51 levels in the mutants (N>11); (***) P < 5 × 10^−4^, t-test. (C) Localization of Myo52-tdTomato in homothallic wildtype and *myo51*Δ strains. (D-E) Measurements of Myo52-tdTomato zone width (D) and fluorescence intensity (E) at the cell-cell contact site in strains as in (C) (N = 15). (F) Quantifications of the number of Myo52 dots observed in *myo51*Δ mutants in comparison to wildtype (N>100 cells). (G) Fusion efficiency of *h90* wildtype, *myo52*Δ, *myo52*Δ *rng8*Δ, *myo51*Δ and *myo51*Δ *rng8*Δ strains on pad (N>200); (*) P < 0.04, (**) P < 0.003 t-test. (H) Localization of Rng8-mEGFP in *myo51Δ* and *myo52Δ* mutants during vegetative growth (top) and during mating (bottom) in *h+* cells crossed to a wildtype *h-* strain expressing Myo52-tdTomato. (I) Measurements of average Rng8-mEGFP fluorescence at fusion site in strains as in H (N = 10). (J) Measurements of Rng8-mEGFP total zone width at the cell-cell contact site in strains as in (H) (N = 10); (***) P < 7 × 10^−5^, t-test. Bars, 2 μm.

Similar to the situation during cytokinesis [21], Myo51 localization at the fusion focus was strongly reduced, though not completely abolished, in *rng8Δ* and *rng9Δ* cells (Fig 4A and 4B). In addition, *myo51Δ* cells are partly fusion defective and strongly fusion incompetent when mated with *fus1Δ* partners [7], similar to *rng8Δ* and *rng9Δ* cells. However, in contrast to *rng8Δ* and *rng9Δ* cells, the Myo52-labelled fusion focus was not significantly broader in *myo51Δ* than in wildtype cells (Fig 4C–4E). In addition, the vast majority of *myo51Δ* cells formed a single Myo52 dot, with only about 30% forming ≥2 dots (Fig 4F). While this is significantly different from the wildtype situation, where about 15% of cells are observed with ≥2 dots, this does not recapitulate the *rng8/9Δ* phenotype where about 95% of cells form ≥2 dots. These data indicate that the fusion focus clustering defect of *rng8/9* mutants is not solely due to a loss of Myo51 function. In agreement with this, *rng8Δ* and *myo51Δ* showed additive phenotypes in fusion efficiency, with the double mutant significantly less fusion-competent than either single mutant. Expectedly, *rng8Δ* was also additive with *myo52Δ* (Fig 4G). Similar results were observed with the *rng9Δ myo51Δ* double mutant (S4 Fig). Rng8 localization was also significantly broader, though not weaker, at the fusion site in *myo51Δ* (Fig 4H–4J), suggesting that one role of Myo51 myosin is to concentrate the Rng8/9 dimer in the fusion focus. In conclusion, the myosin V Myo51 and the Rng8/9 dimer each have independent function during fusion and mutually contribute to concentrate the other on the fusion focus.

### A tropomyosin point mutant recapitulates the *rng8/9Δ* phenotype

Recent in vitro work has shown that the Rng8/9-Myo51 complex binds tropomyosin-decorated actin filaments independently of the Myo51 motor domain [22]. This binding was proposed to anchor the complex to tropomyosin-decorated filaments to favor their transport along other actin filaments bound by the motor domain. This prompted us to examine the role of tropomyosin Cdc8 in actin focus formation.

**Fig 5:**
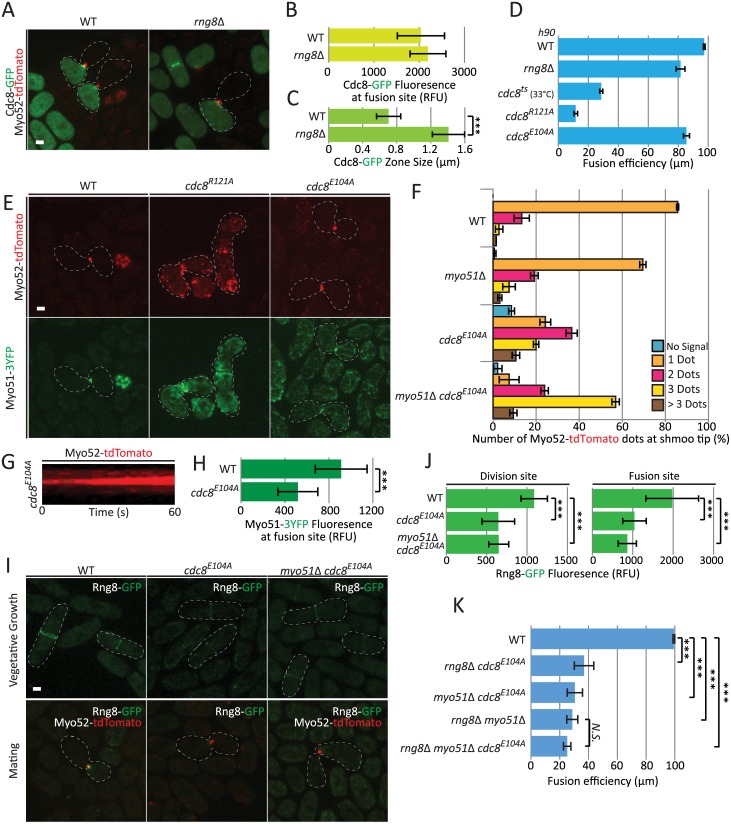
A tropomyosin point mutant recapitulates the *rng8/9* deletion phenotype. (A) Cross of wildtype *h+ myo52-tdTomato* with *h-* wildtype or *rng8*Δ cells expressing nmt41-Cdc8-GFP. (B) Measurements of Cdc8-GFP accumulation at fusion site (N = 15). (C) Measurements of Cdc8-GFP zone width at the cell-cell contact site (N = 10); (***) P < 10^−7^, t-test. (D) Fusion efficiency of *h90* wildtype, *rng8*Δ, *cdc8-382*, *cdc8*^*R121A*^ and *cdc8*^*E104A*^ mutants on pads (N>600). Note that all strains were incubated at 30°C except for *cdc8-382*, which was incubated at the semi-restrictive temperature of 33°C. (E) Localization of Myo52-tdTomato and Myo51-3YFP in *h90 cdc8*^*R121A*^ and *cdc8*^*E104A*^ mutants. (F) Quantifications of the number of Myo52-tdTomato dots observed in *cdc8*^*E104A*^ *and cdc8*^*E104A*^ *myo51*Δ mutants in comparison to wildtype (N>100 cells). The quantification of Myo52-tdTomato dots in *myo51Δ* mutants is identical to Fig 4F and shown for comparison. (G) Kymographs of Myo52-tdTomato showing multiple stable dots over >60s in *cdc8*^*E104A*^ mutant. (H) Myo51-3YFP fluorescence at the fusion site is decreased in *cdc8*^*E104A*^ mutant (N = 15); (***) P < 3 × 10^−5^, t-test. (I) Localization of Rng8-mEGFP in *h+ cdc8*^*E104A*^ and *myo51*Δ *cdc8*^*E104A*^ during vegetative growth and when crossed to *h-* Myo52-tdTomato wildtype cells during mating. (J) Measurements of Rng8-mEGFP fluorescence at the division and the fusion sites show a significant reduction in the *cdc8*^*E104A*^ and *myo51*Δ *cdc8*^*E104A*^ mutant allele (N = 15); (***) P < 1.7 × 10^−8^, t-test. (K) Fusion efficiency of wildtype, *rng8*Δ *cdc8*^*E104A*^, *myo51*Δ *cdc8*^*E104A*^, *rng8*Δ *myo51*Δ *cdc8*^*E104A*^ and *rng8*Δ *myo51*Δ (N>150); (***) P < 6 × 10^−5^, t-test. Bars, 2 μm.

Cdc8 was previously shown to be necessary for cell fusion and to localize at the fusion site [11]. Cdc8-GFP, expressed under the inducible *nmt41* promoter [39], indeed accumulated at the fusion site in both wildtype and *rng8Δ* cells to similar levels, though it occupied a zone about twice as wide in *rng8Δ* cells, as described above for other fusion focus components (Fig 5A–5C). We also confirmed that cells of the temperature-sensitive *cdc8-382* mutant [40], though able to form pairs, were highly fusion-deficient at the semi-permissive temperature of 33°C (Fig 5D). These data confirm an important role of tropomyosin in cell fusion.

We then used a collection of point mutations in predicted surface-exposed Cdc8 residues conserved in fungi [41,42] to screen for non-conditional mutants that would hinder cell fusion when homothallic. This identified two alleles each carrying a single point mutation, *cdc8*^*R121A*^ and *cdc8*^*E104A*^, with reduced fusion efficiency (Fig 5D). The phenotype of *cdc8*^*R121A*^ was very severe, with only about 10% successful fusion. The *cdc8*^*R121A*^ mutation causes significant actin cytoskeleton organization defects during vegetative growth, including weak actin cables, dispersed patches and defective cytokinetic ring, and reduces the affinity of tropomyosin for actin about 30-fold in vitro [42]. During mating, both Myo52-tdTomato and Myo51-3YFP failed to concentrate at a single focal point at the fusion site in this strain, though they were enriched at the zone of cell-cell contact, strongly suggesting that the global organization of the actin cytoskeleton is affected and the fusion focus does not form. We conclude that the fusion defect observed in *cdc8*^*R121A*^ cells is due to strongly reduced actin-tropomyosin interaction.

The second fusion-defective allele, *cdc8*^*E104A*^, displayed a much milder fusion problem, similar to that observed in *rng8Δ* (Fig 5D). This allele was shown to have some very mild cell polarization defects during vegetative growth, but does not affect actin binding [42]. Remarkably, the localizations of Myo52 and Myo51 during mating strongly resembled those observed in *rng8Δ* cells: most *cdc8*^*E104A*^ cells exhibited 2 or 3 Myo52 dots that were spatially stable at the cell-cell contact site (Fig 5E–5G), though we note the phenotype was not quite as severe as that of *rng8Δ* (see Fig 3D). In addition, Myo51 was present in significantly reduced amounts (Fig 5H). The similarity of the *cdc8*^*E104A*^ and *rng8Δ* phenotypes suggests that *cdc8*^*E104A*^ affects Rng8/9 function. Indeed, Rng8 was strongly delocalized from all actin structures: it could not be detected on actin cables and only weakly on the cytokinetic ring during vegetative growth, as well as on the fusion focus during mating (Fig 5I and 5J). As observed in wildtype background, we note that this localization was not further weakened by deletion of Myo51 (Fig 5I and 5J). These results suggest that the fusion defects observed in *cdc8*^*E104A*^ stems from a loss of binding with the Rng8/9 dimer.

Two pieces of data suggest that interaction of the Rng8/9 dimer with both tropomyosin and myosin V Myo51 contribute to focalization of the fusion focus. First, construction of a double mutant *cdc8*^*E104A*^ *myo51Δ* exhibited more severe de-clustered focus phenotype than either single mutant (Fig 5F), identical to *rng8Δ* (see Fig 3D). Second, epistasis analysis showed that the triple *cdc8*^*E104A*^ *myo51Δ rng8Δ* mutant was not more fusion-defective than the double *myo51Δ rng8Δ* mutant, suggesting the *cdc8*^*E104A*^ mutation does not affect other components than Rng8 and Myo51 (Fig 5K). By contrast both *cdc8*^*E104A*^ *rng8Δ* and *cdc8*^*E104A*^ *myo51Δ* double mutants were significantly more fusion-defective than the corresponding single mutants (Fig 5K, compare to Figs 4G and 5D), suggesting the *cdc8* mutant weakens the interaction of both Rng8/9 and Myo51 with actin filaments sufficiently to abolish the function of the complex. We conclude that Rng8/9 acts through both tropomyosin and myosin V Myo51 to organize the fusion focus.

## Discussion

### The homothallic deletion collection: A new genetic tool

Systematic gene deletion collections in both budding and fission yeasts have enabled important advances in the understanding of fundamental cellular processes [28,43,44]. To facilitate the discovery of genes with function in the sexual reproduction process, we derived a self-fertile (homothallic) version of the collection of viable deletions in fission yeast. Because both partner cells carry the same deletion, this approach is more sensitive in identifying genes important for the mating process, whose presence in one of the two partners may be sufficient for functionality. This allowed the discovery of >200 genes involved in cell-cell fusion, a process previously noted for its robustness [4].

This approach also ensured that diploid zygotes were homozygote mutant, leading to the discovery of sporulation-deficient mutants. A similar strategy, using a homothallic derivative of the deletion collection to screen for sporulation-defective mutants through absence of iodine staining, which specifically stains spores, was published during the course of our work [27]. Our list of sporulation-defective mutants overlaps with that described in this work, but is more extensive (S4 and S5 Tables), likely because visual screening permitted identification of more subtle phenotypes, for instance of abnormal spore number.

Besides these two large phenotypic classes, a large number of deletions strains were identified with defect in cell polarization, and categorized in several phenotypic classes. Cell polarization in response to pheromone, leading to cell-cell pairing, is a complex process involving an exploratory patch of active Cdc42 GTPase that serves as site of pheromone release and signaling [5,6]. We note that genes involved in cell polarization during vegetative growth, though present in the deletion collection, were not prominent the *shmoo shape defects* class, suggesting that regulatory mechanisms of polarized growth are in part distinct, as also previously suggested [45]. Mutants with aberrantly placed shmoos, absent from cell sides, or formed in absence of a partner may be caused by a defect in the Cdc42 exploratory polarization mechanism, or may reflect an alteration in pheromone signaling or perception, which modulates exploratory polarization [5,6]. Our screen also identified a large number of mutants with a decreased mating efficiency or forming multiple septa. These two categories were not further investigated and we cannot exclude that some of these mutants have general defects in cell growth and division rather than specifically in the sexual lifecycle.

Finally, one unexpected and very interesting category of mutants is the *promiscuous* class. While wildtype cells always mate with a unique partner, yielding diploid zygotes, these mutants showed multiple cell projections to several partner. While time-lapse microscopy will be required to ascertain whether cells shmoo in all direction at the same time or sequentially, and whether they fuse or only attempt to with several partners, we confirmed that deletion of the master regulators of meiosis *mei2* and *mei3* [46–48] show successive fusion with multiple partners. This phenotype was so extensive in the screen that asci were not readily identified and thus the absence of spores was missed. The mere existence of this category of mutants indicates the existence of regulatory mechanisms that arrest mating in zygotes and thus ensure the alternation of haploid and diploid generations (A. Vjestica, LM and SGM, manuscript in preparation).

In summary, our visual screening of a homothallic derivative of the collection of viable gene deletions exposes a host of novel gene functions that begin revealing new biology and provides a rich basis for future research. This homothallic deletion collection also represents a novel resource that can be further screened for more specific phenotypes.

### Fusion-deficient mutants: Commonalities for fusion in ascomycetes

We focused on the class of fusion-defective mutants, which represents the largest well-defined phenotypic class. The identified mutants may affect any of the multiple steps required to achieve cell-cell fusion, from signaling, cell-cell adhesion, cytoskeletal organization, cell wall digestion to plasma membrane fusion. We note that no other deletion than *fus1Δ* showed a fully penetrant phenotype. This may be due to three main reasons. First, there is significant redundancy between components and/or pathways, as also noted in the study of cell-cell fusion in budding yeast and *Drosophila* myoblasts [4]. For instance, neither Myo51 nor Myo52 is essential for fusion, yet double deletions fully abrogate it [7]. Second, some components may be re-used several times during the mating process, such that their deletion blocks mating at an earlier stage than fusion. This is for instance the case of the pheromone-MAPK cascade, essential for sexual differentiation, but which re-localizes to the fusion focus to signal fusion commitment [10]. Finally, fusion may rely on components otherwise essential for viability, which could not be identified in this screen. For instance, fusion requires a dedicated actin structure, the fusion focus, which, besides its formin nucleator Fus1, is built from components also necessary during cell division [7,11,12]. However, this screen provides a very large entry-point into the fusion process.

It is interesting that the homologues of several genes or pathways required for cell fusion in *S*. *cerevisiae* were identified as fusion-defective in our screen (Fig 2B). These include in particular the BAR adaptor Hob3, which binds the Cdc42 guanine nucleotide exchange factor Gef1 and helps promotes GTP exchange on Cdc42 [37], and Gef1 itself. The *S*. *cerevisiae* homologue of Hob3, Rvs161p, regulates fusion through interaction with Fus2p [49]. While Fus2p has no identifiable sequence homolog in *S*. *pombe*, it directly binds active Cdc42p, and both Cdc42p and its guanine exchange factor Cdc24p are required for fusion [50,51]. The Cdc42 GEF Bud3p also contributes to cell fusion in *S*. *cerevisiae* [52,53]. We also found that the PAK kinase Shk2 is required for fusion, arguing that a common set of proteins around Cdc42 regulates cell fusion in both organisms. Similarly, the deletions of Tea1 and Tea4, important regulators of cell polarity delivered to cell poles by microtubules during mitotic growth [54–56], are present in the fusion-defective class. In *S*. *cerevisiae*, the homologue of Tea1, Kel1p, promotes cell fusion through regulation of Fus2p localization [57,58]. Finally, we identified one of the M-factor-coding genes *mfm1* in the fusion-defective class. This is consistent with the notion that fusion commitment in *S*. *pombe* requires a sharply graded pheromone signal [10], and similar to findings *S*. *cerevisiae* where repression of *mfa1*, coding for *a*-factor, or mutation of its transporter lead to cell fusion defects [59,60]. Finally, as noted previously, formin activities (Fus1 in *S*. *pombe* and likely Bni1 in *S*. *cerevisiae*) and the multi-pass transmembrane protein Prm1 are required for fusion in both species [4,9,23,24,61]. Together, these findings suggest that the process of cell-cell fusion is likely to be highly conserved between these two distant ascomycete species.

### Rng8 and Rng9 are required for fusion focus clustering before stabilization

The phenotype of *rng8Δ* and *rng9Δ* is distinct from previously reported phenotypes: the fusion focus is partly de-clustered, yet each dot is spatially stable and appears to accumulate pheromone-signaling components. Formation of the fusion focus in wildtype cells initiates from a broad distribution of Myo52 at the cell projection cortex, which coalesces into a single focus [7]. Intermediate multi-dots stages resembling the *rng8Δ* phenotype can be transiently observed, but the small clusters are not maintained over time and immobilization happens only for a single structure. We suggest that Rng8/9 normally acts before fusion focus stabilization to ensure the formation of a singular actin aster.

The outcome of the de-clustered focus is that cell wall digestive enzymes are not released at a single location. In the wildtype situation, cell wall hydrolytic enzymes (glucanases) are released specifically at the fusion focus, while glucan synthases are broadly localized, yielding a probable gradient of cell wall hydrolytic activity [7]. When the fusion focus is de-clustered, this gradient likely cannot be well established. Consistently, the glucanase Exg3 was difficult to detect. The consequence is that in crosses to *fus1Δ*, *rng8Δ* cells are largely unable to overcome the homogeneous release of hydrolytic enzymes by their partner, and thus fusion fails. By contrast, when mated to wildtype or itself, *rng8Δ* cells often succeed in cell wall digestion, likely because there is one dominant focus.

### Rng8/9 clusters the fusion focus through tropomyosin interaction

The Rng8/Rng9 complex has emerged as an important regulator of the type V myosin Myo51 [21,22]. Myo51 is an unusual myosin V: in vitro work has shown it is largely monomeric, has a low duty-ratio and is unable to move continuously on actin as a single molecule [22,62]. Similarly, dim punctae of Myo51 (thought to represent dimers) do not move processively on actin cables in vivo [21]. However, assemblies of several Myo51 molecules display processive movements both in vivo and in vitro. Two hypotheses have been proposed for the role of Rng8/9. Rng8 and Rng9 co-purify as oligomers from cells and these proteins convert non-processive Myo51 punctae into processive larger assemblies in vivo. Thus a first model is that Rng8/9 converts Myo51 into a processive motor through cluster formation [21]. Recent in vitro work has shown that Rng8/9 also provides an ATP-independent binding site for the Myo51-Rng8/9 complex to bind tropomyosin-decorated actin, independently of the Myo51 motor domain. This immobilizes the complex when bound to a single filament, but promotes filament bundling or sliding, depending on assay conditions, when two distinct filaments are connected [22]. Thus, a second hypothesis is that Rng8/9 anchors Myo51 to a neighboring actin filament, in a tropomyosin-dependent manner, to favor filament bundling and/or sliding.

Our data lend strong in vivo support for the importance of the Rng8/9-tropomyosin interaction in the assembly of the fusion focus. Tropomyosin was known to be critical for cell fusion [11], and we have confirmed, through use of the *cdc8*^*R121A*^ allele, which displays 30-fold lower actin binding [42], that tropomyosin-actin binding is indeed essential. We now show that *rng8* deletion and *cdc8*^*E104A*^, a tropomyosin point mutant that strongly compromises Rng8 localization to actin structures but does not affect actin binding (our data and [42]), yield almost indistinguishable phenotypes in fusion focus de-clustering. These data predict that the highly conserved region around tropomyosin E104 [42] serves as specific binding site for Rng8/9, though this will need to be confirmed through in vitro reconstitution studies. We note that the additive phenotype of the *rng8Δ cdc8*^*E104A*^ double mutant suggests that this region on tropomyosin also plays a role in Myo51 binding. Because Rng8 localization to actin structures was also strongly compromised in *cdc8*^*E104A*^ vegetative cells, it will be interesting to investigate the possible cytokinetic defects and epistasis of *cdc8*^*E104A*^ in comparison to *rng8Δ*, to generalize these findings to all actin structures.

By contrast, the interaction between Rng8/9 and Myo51 appears less critical for fusion focus organization. Myo51 likely plays a small role, but its deletion shows only very weak de-clustering phenotype and is strongly additive to *rng8Δ* in terms of fusion efficiency. In addition, the observation that Rng8 fails to be enriched on the fusion focus in *myo51Δ* cells suggests the prime function of the Myo51-Rng8/9 interaction during fusion may be to concentrate Rng8/9 at the fusion site. We conclude that Rng8/9 binding to tropomyosin-decorated actin is critical to focus the actin fusion structure.

The fusion focus may be in some ways considered an analogous actin-based structure to the microtubule-based mitotic spindle pole. Spindle pole focusing strongly depends on minus-end directed motor proteins [63], but also of non-motor microtubule-associated proteins. In particular, the non-motor spindle matrix protein NuMA has activities very analogous to those of Rng8/9 in spindle pole focusing: NuMA forms dimers or oligomers, and binds both the pole-directed dynein complex and microtubules directly. Thus, it may focus spindle poles through two possible scenarios: by forming a dynein-NuMA complex that provides two MT binding sites to cross-link and slide MTs passed each other or through NuMA oligomers that directly cross-link MTs [64–66].

Our data suggests that the Rng8/9 complex functions in the fusion focus much like NuMA at the spindle pole. With membrane-proximal Fus1 nucleating actin filaments that are decorated by tropomyosin, Rng8/9-tropomyosin interaction may promote filament-filament interactions and focus formation in two complementary ways. Formation of a complex with Myo51 may allow concentration of Rng8/9 and sliding of filaments (as proposed in [22]) towards the membrane-proximal barbed end. This would contribute to the coalescence of actin filaments to a single focal point, though our data suggest this contribution is modest. Alternatively, and likely more prominently, Rng8/9 may form oligomeric assemblies that crosslink tropomyosin-decorated actin filaments in absence of motor. As oligomers were not detected in vitro [22], their formation may be indirect or require specific post-translational modification. Cross-linking of filaments may selectively stabilize these filaments, thus leading to progressive structure focalization. The Rng8/9-dependent mode of fusion focus clustering may represent one of several mechanisms. Future study of the here-identified collection of deletion promises to reveal fundamental mechanisms of cytoskeletal organization and cell fusion.

## Materials and methods

### Yeast strains and culture

Strains used in this study are listed in S6 Table. For assessing exponentially growing cells, cells were grown in Edinburgh minimal medium (EMM) or minimal sporulation media with nitrogen (MSL+N) supplemented with amino acids as required. For assessing mating cells, liquid or agar minimal sporulation media without nitrogen (MSL-N) were used [38,67]. All live-cell imaging was performed on MSL-N agarose pads [38]. Mating assays were performed as in [5,7,38]. Briefly, pre-cultures of cells were grown at 25°C to OD600 = 0.4–1 in MSL + N (for heterothallic crosses, cells were mixed in equal parts), diluted and grown for 18–20 h to OD600 = 0.4–0.6 at 30°C in MSL + N. Cells were pelleted by centrifugation and washed three times in MSL-N and mounted onto MSL-N 2% agarose pads and sealed with VALAP. Pads were then incubated for either 1 h at 25°C before imaging in overnight movies or overnight at 18°C before imaging. Fusion efficiency was measured as in [7,10].

### Genetic screen

The haploid *S*. *pombe* deletion mutant library was purchased from Bioneer (South Korea). The deletion strains are marked with a G418-resistance *kanMX* cassette in an *h+* strain background (*h+ ade6-M210 ura4-D18 leu1-32*). To examine phenotypic changes during mating, an *h90* library was created by crossing the collection of deleted mutants with a homothallic strain carrying a Nat-resistance *natMX* cassette at the *h90* locus (YSM2945 *h90 mag2-natMX-rpt6*). This strain was made by a PCR based approach using primers osm1023 (5’-caacaagagctgcgttgactgctttttttttgctatataatccagatgcagattattttaaaatactaatccaaatatCGGATCCCCGGGTTAATTAA) and osm1024 (5’ttaatgggttgtttgtcagtcgttgatttagtcctgaatatacataaggaaaagttaatccagggtggagtcgactctGAATTCGAGCTCGTTTAAAC-) to amplify the natMX cassette from pFA6a-NatMX6. This product was designed to recombine into the intergenic region between the mag2 and rpt6 open reading frames at the mat locus (homology is underlined).

Before performing the phenotypic analysis, the collection was amplified and frozen down at -80°C. For amplification, the deletion strains were inoculated in 200μl MSL+N in 384-well plates with the help of a Tecan robot and incubated at 30°C with shaking for 2 days. Pre-cultures of *h90 mag2-natMX-rpt6* cells were grown at 25°C to OD600 = 0.4–1 in MSL+N, diluted and grown for 18–20 h to OD600 = 0.4 at 30°C in MSL+N. 25μl of each deletion strain cultures were mixed with 25μl of the *h90* strain in a 384-well plate and 2μl of the mixture were spotted on EMM-ALU plates containing low nitrogen amounts (24mM NH_4_Cl) with the help of a Tecan robot. Plates were incubated at 25°C for 4 days to allow mating and sporulation, and then shifted to 42°C for 4 days to kill un-sporulated diploid and un-mated haploid cells. The EMM-ALU plates were replica plated on YE with the help of a Singer robot and incubated for 2 days at 30°C to allow spore germination and colony growth. YE plates were replica plated on YE plates containing both G418 and nourseothricin (250μg/ml G418, 100μg/ml Nat) and incubated for 2 days at 30°C to select for *h90* deletion strains. To freeze down the collection, mutants were inoculated from YE-G418/Nat plates in 200μl YE in 96-well plates with the help of a Tecan and a Singer robot. Cells were grown at 30°C for 2 days and 100μl YE containing 50% glycerol was added with the help of a Tecan robot before freezing down the strains at -80°C.

For phenotypic analysis of *h90* deletion strains, the homothallic mutants were first spotted on YE and growth at 25°C for 2 days, and then replica plated on MSL-N and incubated at 25°C for 2 days. Mutants were visually screened on a small table-top Leica microscope with 40x magnification for mating defects. Practically, cells were picked up with a toothpick and resuspended in 2μl MSL-N on a glass slide and coated with a coverslip. The analysis was done in duplicate by 2 independent investigators and phenotypic defects were classified and scored between 1 and 10. Mutants with score ≥ 5 were screened a second time to confirm the phenotypic defect. We note that diploid killing during *h90* collection generation was largely efficient as we observed a few azygotic tetrads (issued from the sporulation of a diploid, rather than a freshly formed zygote) in only 76 strains through the entire visual screen.

GO enrichments were performed using GO term finder (http://go.princeton.edu/cgi-bin/GOTermFinder

### Microscopy and image analysis

The spinning-disk microscope system, previously described [16] was used throughout the study. Optical slices were acquired every 0.6 μm, and all panels show maximum projections, unless otherwise indicated. For zone size measurements, fusion efficiency and number of Myo52 dots at fusion site (Figs 2D, 3B, 3D, 4B, 4C, 4F, 4I, 5C, 5D, 5F, 5G and 5K), the plugin ObjectJ in ImageJ (National Institutes of Health) was used. Fluorescence intensities of Myo51-GFP, Rng8-GFPand nmt41-cdc8-GFP in Figs 3B, 4D, 4G, 5B, 5I and 5J were measured in ImageJ using a manually drawn area around the shmoo tip in sum projections of seven slices over 4-μm total depth. Background fluorescence was measured and subtracted from the original measurements. Kymographs in Figs 3C and 5H were constructed in ImageJ version 1.47 (National Institutes of Health) by drawing a 3-pixel-wide line at the cell tip. Fig were assembled with Adobe Photoshop CS5 and Adobe Illustrator CS5. All error bars are standard deviations. All experiments were done a minimum of three independent times, and statistical analysis was done across repeats of the same experiment.

## Supporting information

S1 Fig Rng8 and Rng9 co-localize with Myo52 at the fusion focus. Maximum intensity projection images of *h90* strains expressing Myo52-tdTomato and either Rng8-mEGFP or Rng9-mEGFP. Bars, 2 μm.
(EPS)

S2 Fig Multiple Myo52 dots observed in wildtype cells during fusion focus maturation. Typical kymographs of Myo52-tdTomato showing multiple unstable dots during the transition from the broad, crescent-like distribution of Myo52 (left) to the formation of a single focus (middle). Once the focus is formed, only a single structure is observed (right). Bars, 2 μm.
(EPS)

S3 Fig The fusion defect of *rng8Δ* and *rng9Δ* is not additive. Fusion efficiency on pad of wildtype and *rng8Δ* and *rng9Δ* single and double mutants as homothallic and when crossed to *fus1*Δ (N>200).
(EPS)

S4 Fig The fusion defects of *rng9Δ* and *myo51Δ* are additive. Fusion efficiency of *h90* WT, *myo52*Δ, *myo52*Δ *rng9*Δ, *myo51*Δ and *myo51*Δ *rng9*Δ on pad (N > 200); (*) P < 0.02, (**) P < 0.005 t-test.
(EPS)

S1 Table Description of the phenotypic classes and other features recorded in the primary screen of the *h90* collection. (XLSX)

S2 Table Screen results. All deletion strains screened are present in the table, with their score for each of the recorded phenotypes from the primary screen (from 1 to 10). In all cases, the score 10 indicates a very penetrant phenotype (but not always fully penetrant) and 1 indicates a weak or low-penetrance phenotype. When the phenotype was only recorded by one of the two investigators, that phenotype is marked with an asterisk. This often happens in deletion strains with apparent low mating efficiency, in which only few mating cells could be observed. When phenotypes were recorded by both investigators, the score represents the average of the two individual scores. The meaning of the score '0' depends on the phenotypic class, as indicated on the right of the table. All recorded phenotypic classes are described in S1 Table.
(XLSX)

S3 Table Fusion-defective phenotypic class. The score 10 indicates a very penetrant phenotype (but not always fully penetrant) and 1 indicates a weak or low-penetrance phenotype. When the phenotype was only recorded by one of the two investigators, that phenotype is marked with an asterisk. This often happens in deletion strains with apparent low mating efficiency, in which only few mating cells could be observed. When phenotypes were recorded by both investigators, the score represents the average of the two individual scores.
(XLSX)

S4 Table Sporulation-defective class. The score 10 indicates a very penetrant phenotype (but not always fully penetrant) and 1 indicates a weak or low-penetrance phenotype. When the phenotype was only recorded by one of the two investigators, that phenotype is marked with an asterisk. This often happens in deletion strains with apparent low mating efficiency, in which only few mating cells could be observed. When phenotypes were recorded by both investigators, the score represents the average of the two individual scores. Some deletion strains were found to have asci with <4 spores, which are marked here by 'low count’.
(XLSX)

S5 Table Comparison of the sporulation-defective class with genes known to be involved in sporulation in *S*. *pombe* prior to this screen or identified in Ucisik-Akkaya et al, 2014. (XLSX)

S6 Table List of strains used in this study. (DOCX)
