# Supplementary material for: A systematic screen for morphological abnormalities during fission yeast sexual reproduction identifies a mechanism of actin aster formation for cell fusion
Source: PLoS Genet. 2017 Apr 14;13(4):e1006721. doi: 10.1371/journal.pgen.1006721 (PMC5409535; doi:10.1371/journal.pgen.1006721)
Supplement: S6 Table — (DOCX) [file pgen.1006721.s010.docx]

**Supplementary Table 6. Strains used in this study**

| Figure 1 | | |
| --- | --- | --- |
| YSM2954 | *h90 mag2-nat-rpt6 ade6- leu1-32 ura4-D18* | This Study |
| Figure 2 | | |
| YSM1396 | *h90 WT (968)* | Lab Stock |
| YSM1442 | *h90 fus1::LEU2 ura4-D18* | [[1](#_ENREF_1)] |
| YSM2955 | *h90 acp2::KanMX6 myo52-tomato-natMX6 pmap3-GFP-ura4+ ade6- leu1-* | This Study |
| YSM2956 | *h90 slm1::KanMX6 myo52-tomato-natMX6 pmap3-GFP-ura4+ ade6- leu1-* | This Study |
| YSM2957 | *h90 twf1::KanMX6 myo52-tomato-natMX6 pmap3-GFP-ura4+ ade6- leu1-* | This Study |
| YSM2958 | *h90 rng8::KanMX6 myo52-tomato-natMX6 pmap3-GFP-ura4+ ade6- leu1-* | This Study |
| YSM2959 | *h90 rng9::KanMX6 myo52-tomato-natMX6 pmap3-GFP-ura4+ ade6- leu1-* | This Study |
| YSM2960 | *h+ acp2-GFP-kanMX6 ade6-M210 leu1-32 ura4-D18* | [[2](#_ENREF_2)] |
| YSM2961 | *h- acp2-GFP-kanMX6 ade6-M210 leu1-32 ura4-D18 his7-* | [[2](#_ENREF_2)] |
| YSM2426 | *h- slm1-mEGFP-kanMX6 ade6-M210 leu1-32 ura4-D18* | [[3](#_ENREF_3)] |
| YSM2962 | *h+ slm1-mEGFP-kanMX6 ade6-M210 leu1-32 ura4-D18* | This Study |
| YSM2428 | *h- twf1-GFP-kanMX6 ade6- leu1-32 ura4-D18 his7-* | [[2](#_ENREF_2)] |
| YSM2963 | *h+ twf1-GFP-kanMX6 ade6- leu1-32 ura4-D18* | This Study |
| YSM2422 | *h- rng8-mEGFP-kanMX6 ade6-M210 leu1-32 ura4-D18* | [[4](#_ENREF_4)] |
| YSM2964 | *h+ rng8-mEGFP-kanMX6 ade6-M210 leu1-32 ura4-D18* | This Study |
| YSM2423 | *h- rng9-mEGFP-hphMX6 ade6-M210 leu1-32 ura4-D18* | [[4](#_ENREF_4)] |
| YSM2965 | *h+ rng9-mEGFP-hphMX6 ade6-M210 leu1-32 ura4-D18* | This Study |
| Figure 3 | | |
| YSM1273 | *h+ nmt41-GFP-CHD-leu1+ ade6-M216 ura4-D18* | Lab Stock |
| YSM2966 | *h- rng8::kanMX6 myo52-tomato-natMX6 ade6-M210 leu1-32 ura4-D18* | This Study |
| YSM2967 | *h- rng9::natMX6 myo52-tomato-natMX6 ade6-M210 leu1-32 ura4-D18* | This Study |
| YSM2968 | *h- rng9::natMX6 rng8::kanMX6 myo52-tomato-natMX6 ade6-M210 leu1-32 ura4-D18* | This Study |
| YSM740 | *h+ myo52-tomato-natMX6 ade6-M216 leu1-32 ura4-D18* | Lab Stock |
| YSM2969 | *h- rng8::kanMX6 Fus1-sfGFP-KanMX6 myo52-tdtomato-natMX6* | This Study |
| YSM2970 | *h- rng9::natMX6 Fus1-sfGFP-KanMX6 myo52-tdtomato-natMX6* | This Study |
| YSM2971 | *h- rng9::natMX6 rng8::kanMX6 Fus1-sfGFP-kanMX6 myo52-tomato-natMX6* | This Study |
| YSM2972 | *h- myo52-tdTomato-natMX6 exg3-sfGFP-kanMX6* | This Study |
| YSM2973 | *h- myo52-tdTomato-natMX6 byr1-sfGFP-kanMX6 leu1-32* | This Study |
| YSM2974 | *h- mam1-GFP-natMX myo52-tdTomato-natMX* | This Study |
| YSM2975 | *h- rng8::kanMX6 myo52-tdTomato-natMX6 byr1-sfGFP-kanMX6 leu1-32 ura4-D18* | This Study |
| YSM2976 | *h- rng8::kanMX6 myo52-tdTomato-natMX6 mam1-GFP-natMX6 leu1-32 ura4-D18* | This Study |
| YSM2977 | *h- rng8::kanMX6 myo52-tdTomato-natMX6 exg3-sfGFP-kanMX6 leu1-32 ura4-D18* | This Study |
| Figure 4 | | |
| YSM2515 | *h90 myo52-tomato-natMX6 nmt41::GFP-chd-leu+ ade6- leu1-32 ura4-D18* | [[5](#_ENREF_5)] |
| YSM2978 | *h90 myo51::ura4+ Fus1-sfGFP-kanMX6 Myo52-tdTomato-natMX6 leu1-* | This Study |
| YSM2964 | *h+ rng8-mEGFP-kanMX6 ade6-M210 leu1-32 ura4-D18* | [[4](#_ENREF_4)] |
| YSM2979 | *h+ myo52::ura4+ rng8-mEGFP-kanMX6 ade6-M210 leu1-32* | This Study |
| YSM2980 | *h+ myo51::ura4+ rng8-mEGFP-kanMX6 ade6-M210 leu1-32* | This Study |
| YSM952 | *h- myo52-tomato-natMX ade6-M216 leu1-32 ura4-D18* | Lab Stock |
| YSM2981 | *h+ myo51-3YFP-kanMX6 leu1- ura4-* | Lab Stock |
| YSM2982 | *h+ rng8::kanMX6 myo51-3YFP-kanMX6 ade6-M210 leu1-32 ura4-D18* | This Study |
| YSM2983 | *h+ rng9::natMX6 myo51-3YFP-KanMX6 ade6-M210 leu1-32 ura4-D18* | This Study |
| YSM1396 | *h90 WT (968)* | Lab Stock |
| YSM2544 | *h90 myo52::ura4+ leu1- ura4-* | [[5](#_ENREF_5)] |
| YSM2984 | *h90 myo52::ura4+ Rng8::hphMX leu1-* | This Study |
| YSM2545 | *h90 myo51::ura4+ leu1-* | [[5](#_ENREF_5)] |
| YSM2985 | *h90 myo51::ura4+ Rng8::hphMX leu1-* | This Study |
|  |  |  |
| Figure 5 | | |
| YSM2173 | *h- leu1::nmt41-gfp-cdc8-ura4+* | [[6](#_ENREF_6)] |
| YSM740 | *h+ myo52-tdTomato-natMX6 ade6-M216 leu1-32 ura4-D18* | Lab Stock |
| YSM2986 | *h- rng8::kanMX6 myo52-tomato-natMX leu1::nmt41-gfp-cdc8-ura4+ leu1-32* | This Study |
| YSM2529 | *h90 myo52-tomato-NatMX;myo51-3YFP-kanMX leu1- ura4-* | [[5](#_ENREF_5)] |
| YSM2987 | *h90 cdc8-382 Myo52-tdTomato-natMX Myo51-3YFP-kanMX* | This Study |
| YSM2988 | *h90 cdc8 (R121A)::his5+ura4+ Myo52-tdTomato-natMX Myo51-3YFP-kanMX* | This Study |
| YSM2989 | *h90 cdc8 (E104A)::his5+::ura4+ Myo52-tdTomato-natMX Myo51-3YFP-kanMX* | [[7](#_ENREF_7)] |
| YSM2990 | *h- cdc8 (E104A)::his5+::ura4+ Myo52-tdTomato-natMX* | This Study |
| YSM2991 | *h- cdc8 (E104A)::his5+::ura4+ myo51::ura4+ Myo52-tdTomato-natMX* | This Study |
| YSM2992 | *h+ rng8-mEGFP-kanMX6 myo52-tdTomato-natMX6 ade6-M210 leu1-32 ura4-D18* | This Study |
| YSM2993 | *h+ cdc8 (E104A)::his5+::ura4+ rng8-mEGFP-kanMX Myo52-tdTomato-natMX* | This Study |
| YSM2994 | *h+ cdc8 (E104A)::his5+::ura4+ myo51::ura4+ Myo52-tdTomato-NatMX* | This Study |
| YSM2995 | *h+ cdc8 (E104A)::his5+::ura4+ rng8::kanMX6 Myo52-tdTomato-NatMX* | This Study |
| YSM2996 | *h- cdc8 (E104A)::his5+::ura4+ rng8::kanMX6 Myo52-tdTomato-NatMX* | This Study |
| YSM2997 | *h- cdc8 (E104A)::his5+::ura4+ myo51::ura4+ rng8::kanMX6 Myo52-tdTomato-NatMX* | This Study |
| YSM2998 | *h+ cdc8 (E104A)::his5+::ura4+ myo51::ura4+ rng8::kanMX6 Myo52-tdTomato-NatMX* | This Study |
| YSM2999 | *cdc8 (E104A)::his5+::ura4+ myo51::ura4+ Myo52-tdTomato-NatMX rng8-mEGFP-kanMX6* | This Study |
|  | Supplementary Figures |  |
| YSM3000 | *h- rng9-mEGFP-hphMX myo52-tom-NatMX* | This Study |
| YSM3001 | *h+ rng9-mEGFP-hphMX myo52-tom-NatMX* | This Study |
| YSM2992 | *h+ rng8-mEGFP-kanMX6 myo52-tdTomato-natMX6 ade6-M210 leu1-32 ura4-D18* | This Study |
| YSM3002 | *h- rng8-mEGFP-kanMX6 myo52-tdTomato-natMX6 ade6-M210 leu1-32 ura4-D18* | This Study |
| YSM3003 | *h90 myo52::ura+; Rng9::hphMX leu1- ura4-* | This Study |
| YSM3004 | *h90 myo51::ura+; Rng9::hphMX leu1- ura4-* | This Study |

**Supplementary references**

1. Petersen J, Nielsen O, Egel R, Hagan IM (1998) FH3, a domain found in formins, targets the fission yeast formin Fus1 to the projection tip during conjugation. J Cell Biol 141: 1217-1228.

2. Kovar DR, Wu JQ, Pollard TD (2005) Profilin-mediated competition between capping protein and formin Cdc12p during cytokinesis in fission yeast. Mol Biol Cell 16: 2313-2324.

3. Kabeche R, Baldissard S, Hammond J, Howard L, Moseley JB (2011) The filament-forming protein Pil1 assembles linear eisosomes in fission yeast. Mol Biol Cell 22: 4059-4067.

4. Wang N, Lo Presti L, Zhu YH, Kang M, Wu Z, et al. (2014) The novel proteins Rng8 and Rng9 regulate the myosin-V Myo51 during fission yeast cytokinesis. J Cell Biol 205: 357-375.

5. Dudin O, Bendezu FO, Groux R, Laroche T, Seitz A, et al. (2015) A formin-nucleated actin aster concentrates cell wall hydrolases for cell fusion in fission yeast. J Cell Biol 208: 897-911.

6. Skoumpla K, Coulton AT, Lehman W, Geeves MA, Mulvihill DP (2007) Acetylation regulates tropomyosin function in the fission yeast Schizosaccharomyces pombe. J Cell Sci 120: 1635-1645.

7. Cranz-Mileva S, MacTaggart B, Russell J, Hitchcock-DeGregori SE (2015) Evolutionarily conserved sites in yeast tropomyosin function in cell polarity, transport and contractile ring formation. Biol Open 4: 1040-1051.
